# Toxicological concerns regarding glyphosate, its formulations, and co-formulants as environmental pollutants: a review of published studies from 2010 to 2025

**DOI:** 10.1007/s00204-025-04076-2

**Published:** 2025-05-26

**Authors:** Szandra Klátyik, Gergely Simon, Eszter Takács, Marianna Oláh, Johann G. Zaller, Michael N. Antoniou, Charles Benbrook, Robin Mesnage, András Székács

**Affiliations:** 1 Agro-Environmental Research Centre, Institute of Environmental Sciences, Hungarian University of Agriculture and Life Sciences, Páter K. u. 1, 2100 Gödöllő, Hungary; 2 Pesticide Action Network Europe, Rue de La Pacification 67, 1000 Brussels, Belgium; 3 Department of Integrative Biology and Biodiversity Research, Institute of Zoology, University of Natural Resources and Life Sciences Vienna, Gregor Mendel Straße 33, 1180 Vienna, Austria; 4 Gene Expression and Therapy Group, Faculty of Life Sciences & Medicine, Department of Medical and Molecular Genetics, King’s College London, Guy’s Hospital, London, SE1 9RT UK; 5 Buchinger Wilhelmi Clinic, Wilhelmi-Beck-Straße 27, 88662 Überlingen, Germany; 6 Benbrook Consulting Services, 10526 SE Vashon Vista Drive, Port Orchard, WA 98367 USA

**Keywords:** Glyphosate, Co-formulants, POEA, Microbiome, Oxidative stress, Genotoxicity

## Abstract

Over the last decade and worldwide, an enormous investment in research and data collection has been made in the hope of better understanding the possible ecological and toxicological impacts triggered by glyphosate (GLY). This broad-spectrum, systemic herbicide became the most heavily applied pesticide ever in the 2000s. It is sprayed in many different ways in both agricultural and non-agricultural settings, resulting in multiple routes of exposure to organisms up and down the tree of life. Yet, relatively little is known about the environmental fate of GLY-based herbicide (GBH) formulations, and even less on how GBH co-formulants alter the absorption, distribution, metabolism, excretion, and toxicity of GLY. The environmental fate of GLY depends on several abiotic and biotic factors. As a result of heavy annual GBH use over several decades, GLY residues are ubiquitous, and sometimes adversely affect non-target terrestrial and aquatic organisms. GLY has become a frequent contaminant in drinking water and food chains. Human exposures have been associated with numerous adverse health outcomes including carcinogenicity, metabolic syndrome, and reproductive and endocrine-system effects. Nonetheless, the existence and magnitude of GLY-induced effects on human health remain in dispute, especially in the case of heavily exposed applicators. A wide range of biochemical/physiological modes of action have been elucidated. Various GBH co-formulants have long been considered as inert ingredients relative to herbicidal activity but clearly contribute to GLY-induced hazards and risk gradients. In light of already-identified toxicological and ecosystem impacts, the intensive research focuses on GLY and GBHs should continue, coupled in the interim with commonsense, low-cost changes in use patterns and label requirements crafted to slow the spread of GLY-resistant weeds and reduce applicator and general-population exposures.

## Introduction

The enormous and unprecedented scale of global reliance on glyphosate-based herbicides (GBHs) is responsible for the scope and severity of the documented, possible, and as yet unrecognized adverse effects discussed in this review. Measured by the kilograms of active ingredient (AI) applied, glyphosate (GLY) became the most widely used pesticide ever in the mid-2000s, and both in the United States (US) and globally. The rapid adoption of cotton, soybean, and corn seeds genetically engineered (GE) to tolerate over-the-top applications of GBHs drove the post-1996 meteoric rise in GLY applications (Benbrook 2016). Reliance on GE seeds and GLY rose even more sharply from ~2005 through to peak use in 2015–2017, a period when enough GLY was applied annually to spray around two-thirds of a kilogram of GLY on every cropland hectare in the world. Moreover, the kilograms of GLY applied at peak use likely exceeded the next most widely applied pesticide (atrazine) by more than a factor of two. Based on a European survey, the sales of GBHs were estimated at about 44,250 tons of AI, while the average rate of GLY application was about 0.24 kg AI/ha in 2017 (Antier et al. 2020). The global GLY market was estimated around USD 11.18 billion in 2023 (Zion Market Research 2024). According to another estimate, the GLY market expanded from USD 9.47 billion in 2023 to USD 10.46 billion in 2024 (Research and Markets 2024). However, accurate and up-to-date data on the global use and sales of GBHs are challenging to obtain because of the proliferation of generic manufacturers and the complexity of global supply chains (PAN 2023; Klátyik et al. 2024). As the volume of GBHs applied worldwide rose, the diversity of use patterns and application methods in both the agricultural and non-agricultural sectors also expanded. Over the last four decades, agricultural uses have accounted for around ~ 91% of total global use and non-agricultural uses the remaining ~ 9%. As the diversity of use patterns and application methods expanded, exposure pathways and the frequency of relatively high exposure episodes for both people and non-target organisms also increased. This is why biomonitoring studies conducted on nearly all continents have reported residues of GLY and/or its primary metabolite aminomethylphosphonic acid (AMPA) in most actively farmed soils, water resources, and organisms, including people.

The typical GBHs contain co-formulants crucial to product efficacy. Co-formulants have long been considered as inert, i.e., inactive, relative to the capacity of GLY to control weeds. As a result, registrants, regulators, and many independent researchers have designed studies wholly or mostly focused on just parent compound GLY. Unfortunately, most regulatory assessments and published studies do not assess the significant impacts of co-formulants on product chemistry, environmental fate, absorption, distribution, metabolism, and excretion (ADME), and adverse impacts and outcomes (Mesnage and Antoniou 2018; Székács and Darvas 2018; Fishel 2020; US EPA 2022). This is regrettable because several studies have confirmed the contribution of co-formulants (e.g., mixture of polyethoxylated tallow amines [POEA]) to GBH/GLY exposures and risks, and in particular genotoxicity (Mesnage et al. 2019; Langrand et al. 2020; Maderthaner et al. 2020). Due to the compelling evidence of heightened risk stemming from exposures to POEA-based GBH formulations, the EU Commission banned all GBHs containing POEA surfactants in 2016 (European Commission 2016). Uncertainly persists over the degree to which this action has actually reduced GBH risk levels. Co-formulants are generally present in GBHs at concentrations relative to GLY between 1:4 and 1:7. In addition to hastening the movement of GLY through weed leaf tissues (Defarge et al. 2018), GBH co-formulants also markedly enhance the rate of dermal penetration when spray solution lands on human skin. Co-formulants also accelerate GLY movement through cell walls in the case of dermal or inhalation exposures to spray solution, and can also markedly alter environmental fate.

The two primary sources of human exposure to GLY among the general public not engaged in farming, or living near treated fields, are via drinking water and food (Gillezeau et al. 2019; Solomon 2020). Especially in intensively farmed regions, residues of GLY and AMPA have become ubiquitous in soil and surface waters, and are often present in drinking water (Aparicio et al. 2013; Székács et al. 2015; Silva et al. 2018; Álvarez Bayona et al. 2022; Nunes et al. 2024; Cheng et al. 2025). Over the life history of GLY use and GBH regulation, the primary focus of research and regulatory risk assessments has been the properties and toxicity, and associated risks, following dietary exposure to technical grade GLY (Székács and Darvas 2012; Casida 2017). From commercial introduction in 1974 through 1996, GBHs were used predominately for pre-crop-emergent and/or post-harvest weed control, use patterns that rarely resulting in residues in harvested foodstuffs. Applications of a GBH on a growing crop would kill both weeds and the crop. Post-1996, over-the-top applications of GBHs on genetically modified (GM) crops triggered the explosive growth in GLY kilograms applied in North and South America (Benbrook 2016), although the planting of GM seeds was not authorized in most of Europe.

Pre-harvest crop dessication applications of GBHs emerged as another GBH use pattern in the early 1980s, first in the UK and soon thereafter spreading east through northern Europe, and eventually worldwide. Such uses are also known as “green burndown” applications, which are intended to kill mother plants so that grain and beans will dry down faster and more evenly. This can sometimes allow farmers to begin and complete harvest operations before inclement weather arrives in the fall. Late season rainfall can delay harvest, knock over standing grain crops, and trigger mycotoxin problems (Dill et al. 2010; Klátyik et al. 2023, 2024). Pre-harvest desiccation uses of GBHs account for a very small share of GLY kilograms applied (likely no more than 3% of agricultural uses) but are responsible for the majority of GLY residues in food. A major share of GLY-triggered dietary risk among the general population arises from such applications. For this reason, the EU Commission banned the use of GBHs as pre-harvest desiccants as a part of its renewal of GLY in 2023. One other important adverse impact of pre-harvest GBH applications has received far too little attention. When applied to a mother plant, GLY can translocate into tubers, grain, or beans, rendering them unreliable for use as seed (Bhowmik 1994). This is because the GLY in tubers and seeds, when planted in a subsequent crop year, can become activated as the tubers and seeds begin to grow, leading to serious morphological deformities in the growing crop. Such adverse impacts are known to occur even when GLY is present in seed tubers and grain at very low levels.

Another relatively new use of GBHs is rapidly growing in some farming regions, and especially in the US. The incorporation of cover crops into row-crop production systems has been embraced as a key objective of climate-smart farming, often in conjunction with no-till planting systems. In the US, new policies and subsidies are now dedicated to the promotion of “regenerative” farming systems that utilize no-till and cover crops. In addition, most carbon offset programs and payments to farmers are likewise predominately triggered by farmer agreements to reduce tillage and plant cover crops. While soil carbon sequestration via no-till plus cover crops is both limited and often short-lived (Yi et al. 2025), such “climate-smart” farming systems will increase reliance on GBH applications to kill cover crops. Or even more worrisome in the US, applications of paraquat.

From the mid-1990s through around 2015, adoption of GM seed technology drove the increased use of GBHs. In 2025, the adoption of cover crops is posed to cause another significant, new use of GBHs, but this time advanced by substantial government funding. Even more intense reliance on GBHs in row-crop, GMO-based farming systems will further accelerate the emergence and spread of GLY-resistant weeds, thereby increasing the need for multiple, additional herbicide applications. It will also exacerbate GBH-triggered erosion in soil health, increase GLY water contamination, and cause loss of biodiversity.

Once a GBH is applied, the GLY is metabolized to form its primary degradation products AMPA and sarcosine, accompanied by the release of inorganic phosphorus (Díaz-Soto et al. 2024). The chemical form of GLY and its degradation pathways and dynamics are also affected by several factors including soil and water pH, organic matter content of the soil, farming systems, and soil microbial communities (Li et al. 2025). Microbial degradation of GLY is of particular importance in terms of the environmental concentration and fate of the AI and offers numerous bioremediation possibilities (Morales-Olivares et al. 2025).

The degradation of GLY through the sarcosine pathway involves two primary enzymatic reactions: the hydrolytic cleavage of the GLY molecule to release a phosphate group and produce sarcosine and the oxidation of sarcosine to formaldehyde and glycin (Morales-Olivares et al. 2025). The degradation of GLY via the sarcosine pathway involves two main enzymatic steps: the hydrolytic cleavage of the GLY molecule, which releases a phosphate group and produces sarcosine, followed by the oxidation of sarcosine into formaldehyde and glycine (Morales-Olivares et al. 2025). Once a GBH is sprayed, the GLY and co-formulants almost certainly become separated relatively quickly, but little is known about the environmental fate, or toxicity of co-formulants. The environmental fate and concentration of GLY in different environmental matrices are strongly influenced by soil physical and chemical properties and climatic conditions (e.g., soil composition and structure, pH, and microbial activity). When and how, and how often GBHs and other pesticides are applied, are also key drivers of environmental fate and off-target impacts (Hébert et al. 2019). GLY has been regarded for decades as a relatively safe herbicide in light of its physical and chemical properties and relatively low mammalian toxicity. Both GLY and AMPA were thought to be inactivated in the soil due to adsorption and relatively short soil half-life (Cuhra et al. 2016). But, a growing body of research has reported unexpected movement and persistence in certain regions and environments. Substantial, albeit hard-to-detect adverse impacts on terrestrial and aquatic ecosystems and food chains are known to arise from legally authorized use of GBHs (Hanke et al. 2010; Cederlund 2017).

In particular, heavy use of GBHs over years in some areas has disrupted agro-ecosystems, and/or impaired biodiversity, in ways that do not arise from, nor manifest as direct toxicity and/or observable acute effects. Instead, such adverse effects are triggered by subtle changes in energy flows and non-target organism population dynamics that arise in response to specific biotic and abiotic stressors. The exposure of non-target organisms and wildlife to the residues of GBHs is widespread in different groups or taxa, both in frequently treated agricultural areas and untreated habitats (Fritsch et al. 2025). In short, GLY appears to impose a tax on ecosystem resilience. As GLY loadings increase, the tax on resiliency grows. This is of considerable concern given the widely accepted need for farmers to find new ways to enhance the resiliency of their farming systems in response to climate change and ever-more intense pest pressure.

In the past, GLY has generally not been involved in environmental monitoring programs, thus the concentration of GLY and its metabolites has been unknown or underestimated in various environmental matrices. Periodic surveys conducted by the US Geological Service are an exception (USGS 2020). However, with the improvements in analytical methods, GLY has been recognized as a ubiquitous environmental contaminant (Székács and Darvas 2018; Huhn 2018; Klátyik et al. 2024). Surprisingly, high variability has been observed in residue levels in some environments (Székács and Darvas 2018). AMPA, the primary metabolite of GLY, is much more mobile than the parent compound (Duke and Powles 2008). As a result, it is more frequently detected in various environmental matrices compared to GLY (Chang et al. 2011; Silva et al. 2018; Lutri et al. 2020). As expected, in cropland soils, the level of GLY contamination is strongly correlated with the intensity of agricultural use (Maqueda et al. 2017). GLY and AMPA were present in 45% of 317 topsoil samples from 11 EU countries, where the highest observed concentration was 2 mg/kg (Silva et al. 2018). Based on a recent study, AMPA was detected more frequently under conventional tillage than no-till, with higher persistence observed when combined with either mineral nitrogen fertilization or no fertilization. Conventional tillage seems to influence soil structure and/or bacterial community composition in ways that enhance the degradation and leaching of GLY and AMPA (Petit et al. 2025).

The level of GLY contamination in surface waters can reach up to 5200 µg/L, especially in streams near treated agricultural fields after heavy or sustained rainfall (Edwards et al. 1980; Coupe et al. 2012). Based on environmental monitoring studies, the average reported maximum GLY concentration on the Australian, Asian, American, and European continents in surface waters is approximately 240 μg/L (ppb). However, higher levels have been detected in water resources where GM crops account for a significant share of hectares in production and/or where the use of GLY for desiccation is common (Coupe et al. 2012; Mardiana-Jansar and Ismail 2014; Székács and Darvas 2018; Hénault-Ethier et al. 2019; Campbell et al. 2025). In France, GLY and AMPA are among the most commonly detected compounds in river samples, with quantification frequencies of 43% and 63%, respectively, and concentrations reaching up to 164 μg/L for GLY and 558 μg/L for AMPA (NAIADES 2018). Nonetheless, it is important to highlight that the presence of AMPA and GLY in environmental matrices (e.g., groundwater, wastewater treatment plant effluents, or sewage sludge) may not arise solely from the agricultural use of GBHs and GLY metabolism: the notion has been raised that they may also originate from phosphonate-based detergents and chelating agents used in various cleaning products, household or industrial applications, and during wastewater treatment (Grandcoin et al. 2017; Schwientek et al. 2024; Röhnelt et al. 2025; Tolkamp and Hofman-Caris 2025). The impact from such other identified sources, however, is not anticipated to notably influence the environmental and health risks associated with the total outstanding agricultural GLY emissions. In addition, regulatory maximal residue limits for environmental matrices are established based exclusively on the environmental and health assessment of the toxic effects of the AIs and their biologically active metabolites, irrespective of the source of the pollution. Therefore, from this perspective, it makes no difference whether measurable environmental GLY and AMPA concentrations are introduced into the environment from agricultural practices or other industrial or household applications.

GLY and AMPA were also detected at up to 2.5 and 0.48 μg/L in rain. Levels up to 9.1 and 0.97 ng/m^3^ have been reported in air, respectively, in samples collected from Mississippi, Iowa, and Indiana States (USA) (Chang et al. 2011). The appearance of GLY residues in various environmental compartments can result in negative effects on non-target organisms (Klátyik et al. 2023, 2024; Evalen et al. 2024). Contamination of drinking water and food products can also exceed allowed safety limits (European Parliament and the Council 2002). While nearly ubiquitous in most corners of the environment and in most living creatures, it remains challenging to accurately estimate environmentally relevant concentrations and toxicity-based exposure thresholds. There are many reasons underlying such complexity and challenges. Among them is the fact that GLY levels in the environment, as well as in organisms, are rapidly changing. Levels of exposure are dynamic within organisms. The amount of GLY reaching various tissues varies greatly, and GLY is sometimes immobilized in tissues, including bone (Benbrook 2025). Other key variables include the timing of multiple exposures, the duration of exposure, tissues exposed, and the overlap of exposures with the stage of an organism’s life cycle and its overall health status.

Human exposure to GLY via beverages and food, in addition to environmental pollution, has been associated with numerous adverse health outcomes. GLY residues appear in food and animal feed usually due to preharvest desiccation uses or mid- or late-season applications on GM crops (Cuhra 2015; Myers et al. 2016). Routine human exposure to GLY was demonstrated in independent tests of German beer (Guttenberger and Bär 2016) and US wine, corn, and soybeans (Honeycutt 2016; USDA PDP 2024). The distribution of GLY in the human organism of rural residents due to various sources (e.g., crops, livestock products, air, soil, and water) and routes of exposure (oral, dermal, and inhalation) has been modeled in detail using the internal allocation factor as a main descriptor (Huang and Li 2025). GLY was found in 99% of the French population, a finding almost certainly due to ingestion of food and water bearing GLY residues. In the French study, elevated concentrations were generally detected in urine samples collected from farmers and/or their families living in wine-growing communities. Levels were also generally higher among children and men (Grau et al. 2022). An important US study was conducted within a subcohort of the 83,000 enrollees in the US Agricultural Health Study (Hofmann et al. 2015; Chang et al. 2024). GLY levels were measured in the urine of four groups: (1) non-farming controls (i.e., the general public); (2) long-term farmers with no, or very modest life-long use of GBHs, and no recent uses; (3) long-term farmers with substantial life-long use of GBHs, but no recent use (within the last week); and (4) long-term heavy users who had made a GBH application within one week of the collection of the urine sample. The team reported detectable levels of GLY (≥ 0.2 μg/L) in the urine of 91–93% of the farmers, but also in 81–88% of the non-farm controls (Chang et al. 2024). Significantly, the differences in GLY urine levels were modest between the non-farm controls; farmers who had been heavy users of pesticides, but not GBHs; and long-term users of GBHs who had not made an application in the last week, as shown in Table 1. However, GLY levels were markedly higher among long-term users who had sprayed a GBH within the last week, and still higher among those who had applied a GBH within 1 day of the collection of the urine sample (Table 1). Moreover, urinary GLY levels were found in an epidemiological study associated with glucose dyshomeostasis in a dose-dependent manner, indicating that exposure to GLY may be related as an environmental co-factor in metabolic abnormalities in glucose regulation (Feng et al. 2025).

**Table 1:** Distribution of glyphosate levels in urine in the Biomarkers of Exposure and Effect in Agriculture (BEEA) Subcohort: 2010–2018 (µg/g creatine) (Chang et al. 2024)

	Distribution of GLY levels		
	50 th	95 th	Maximum
Non-farming controls (general population)	0.37	1.08	1.74
Farming controls (no recent use)	0.41	1.1	2.17
High lifetime exposure (no use last 7 days)	0.47	1.64	2.67
GLY applied last 7 days	0.72	7.72	20
GLY applied in last day	2.2		20
	Fold differences		
Applied in last 7 days/non-farming controls	1.95	7.15	11.46
Applied in last day/non-farming controls	5.95	n.a.	11.49

*GLY* glyphosate, *n.a.* not available

In addition to providing key data on the relationship between when a urine sample was collected and GLY levels were measured, Chang et al. (2023a) also reported a significant association between GLY levels and markers of oxidative stress, as measured in the same sample of urine, and furthermore, coupled with accurate data on recent GBH use. In other studies, they identified mosaic loss of chromosome Y from blood samples associated with lifetime occupational use of GLY (Chang et al. 2023b) and minor effects on leukocyte telomere lengths (Erickson et al. 2025). Urinary GLY concentrations negatively correlated with selenium levels in whole blood, indicating that high whole blood selenium levels may increase mortality risks related to GLY (Chu et al. 2025). This is the first and only study we know of that reports such an association based on measurements taken on the same sample of urine. By design, the Biomarkers of Exposure and Effect in Agriculture (BEEA) subcohort substantially reduced the significant exposure-metric error that has plagued other epidemiological studies of GLY and chronic disease carried out within the Agricultural Health Study (AHS) (Zhang et al. 2019; Rana et al. 2023). Latent-class analysis of pesticide use patterns and cancer risk identified heightened risk of colon cancer and pancreatic cancer among enrollees in the AHS (Gerken et al. 2024). Serious concerns have also been raised, particularly about human pregnancy and birth defects among agricultural workers and consumers (Gerona et al. 2022; Parvez et al. 2018), while other studies dismiss such concerns (de Araujo et al. 2016). The main results of toxicological testing on mammals, mammalian, and human cells/cell lines with GLY and/or GBHs are summarized in Table 2.

**Table 2:** Summary of main toxicological studies and effects of glyphosate, its derivatives, co-formulants, and/or its pesticide formulations on mammals, mammalian and human cells/cell lines

Model organism	Test compound	Concentrations	Results	Conclusions	References
JEG3 human cell line (in vitro)	GLY IPA; GBHs (e.g., Roundup WeatherMAX, Glyfos, Roundup Classic); co-formulants (e.g., POEA, quaternary ammonium compound, APG)	Assay- and compound-specific concentration range	All co-formulants and GBHs were cytotoxic below 100 mg/L, cytotoxicity of GLY IPA was not detected; decreased aromatase activity was observed after the exposure to co-formulants (POEA [2.5 mg/L], APG [120 mg/L]), GBHs (25–300 mg/L), and GLY IPA (3000 mg/L)	Improved endocrine disruptive effect of GBHs can be detected in the presence of co-formulants	Defarge et al. (2016)
Granulosa cells of cattle (in vitro)	GLY; GBH (Roundup)	0–300 µg/mL	GBH decreased cell number, estradiol and progesterone production (10 and 300 µg/mL), also in the presence of FSH and IGF-1 (10 µg/mL); in the presence of FSH, GLY increased the estradiol production (10 µg/mL), while the GBH had no effect at the same concentration	GLY alone and especially its formulation (Roundup) has the potential to damage the reproductive functions in cattle	Perego et al. (2017)
Intestinal strips isolated from male Wistar rats (in vitro)	GLY; GBH (Roundup Ultra 170 SL); POEA; mixture of GLY and POEA	GLY: 1.7 g/L; GBH: 0.003–1.7 g GLY/L; POEA: 1.28–800 mg/L; 1.7 g/L GLY and 800 mg/L or 0.051 mg/L POEA in combination	GBH disturbed the motoric activity of the intestine, irreversible effects on the spontaneous contractility and reactivity were detected (GBH: 1.7 g/L GLY equivalent); POEA caused biphasic muscle reaction (relaxation and contraction) at lower concentrations (0.256–6.4 mg/L), while irreversible response was detected at 32–800 mg/L	Higher toxicity of POEA was detected compared to the toxicity of the GBH, antagonistic interaction was assumed between GLY and POEA toward the motoric activity of gastrointestinal tract	Chłopecka et al. (2017)
MCF-7, MDA-MB-231, T47D, and T47D-KBluc cell lines (in vitro)	GLY; GBHs (e.g., Glyphogan, Roundup Grand Travaux Plus, Roundup Original DI); co-formulants (pure POEA, POEA adjuvant formulation)	1–10,000 µg/L	GLY promoted proliferation of MCF-7 human breast cancer cells (10,000 µg/L), increased expression of an estrogen response element-luciferase reporter gene in T47D KBluc cells, and induces changes in gene expression reflective of hormone-induced proliferation in MCF-7 cells; estrogenic effects of GBHs and co-formulants were not detected	High concentration of GLY, but not other ingredients of GBHs, can activate estrogen receptor α in vitro	Mesnage et al. (2017)
Human embryonic kidney 293 cell line (HEK 293, ECACC 85120602) (in vitro)	GLY; GLY IPA; GBHs (e.g., Glyphogan, Glyfos, Roundup Classic); co-formulants (e.g., POEA, quaternary ammonium compound)	Assay- and compound-specific concentration range	GBHs and POEA caused the death of the human embryonic kidney cells within 90 min; cytotoxicity of GLY was not demonstrated at similar equivalent concentrations	Synergistic toxic effects were found indicating the needs for chronic regulatory experiments on the full commercial GBHs to establish the acceptable daily intake of GLY	Defarge et al. (2018)
Human peripheral blood mononuclear cells (in vitro)	GLY; AMPA; GBH (Roundup 360 PLUS)	GLY, AMPA: 0–1000 µM; GBH: 0–50 µM	GBH caused DNA damage even at 5 µM; DNA lesions were induced after the exposure to GLY and AMPA from 250 and 500 µM, respectively	DNA damage induced by GLY and its derivatives in the following order: AMPA < GLY < GBH	Woźniak et al. (2018)
Pregnant mice (in vivo)	GLY; GBH (Roundup)	5 g/L GLY equivalent	Ovarian histopathological alterations, hormonal imbalances, oxidative stress, and interference with the expression of steroidogenesis-related genes were observed in the treated mice	Prenatal exposure to GLY may alter the sex ratios of fetuses; toxic effects of GLY and Roundup on ovary function or steroidogenesis are complex	Ren et al. (2018)
Granulosa, luteal, myometrial and endometrial cells of cow’s ovaries and uteri (in vitro)	GLY; GBH (Roundup)	0.11 and 10 ng/mL	GLY increased the secretion of estradiol from granulosa cells (10 ng/mL); decreased secretion of progesterone and increased oxytocin secretion were detected in luteal cells; the secretion of prostaglandins from endometrial cells was decreased	GLY and GBHs possibly damage the fertilization or may disrupt the maintenance of gestation	Wrobel (2018)
MC3T3-E1 cells (in vitro)	GLY; GBH (Roundup Classic); POEA	Assay- and compound-specific concentration range	Effects on cell morphology were induced in cells exposed to the GBH and POEA; GLY caused late-phase response and affects cell adhesion, mobility, and morphology of the cytoskeleton; cellular effects (e.g., cytoskeletal collapse, cell death) were detected in cells exposed to POEA	Cytotoxicity was higher for POEA compared to GLY and Roundup Classic; no unequivocal correlation between the effects of Roundup Classic and its components	Farkas et al. (2018)
Immature mouse Sertoli cell line (TM4) (in vitro)	GLY; POEA; GBHs (Roundup Bioforce and Glyphogan)	Concentrations ranging from environmental to agricultural-use levels	GBHs induced TM4 mitochondrial dysfunction, disruption of cell detoxification systems, lipid droplet accumulation, and mortality at sub-agricultural doses (10 to 10^4^ mg/L); higher toxicity was proved for POEA compared to the individual toxicity of GLY	Effects induced by GLY and/or formulants should be lower in vivo than in vitro, toxicity of all compounds of GHBs should be studied to conclude on the safety of GBHs, not of GLY alone	Vanlaeys et al. (2018)
Rat (in vivo)	GLY; GBH (Glyfonova)	2.5 and 25 mg/kg bw	Minor effects of the GBH were detected in the exposed rats, with a small upregulation of the steroidogenic genes; no significant effects were observed on testes or testosterone synthesis in rats exposed to GLY alone	Minor effects were demonstrated on steroidogenic gene expression after the GBH exposure probably caused by the presence of co-formulants	Johansson et al. (2018)
MDA-MB-231 and MCF7 breast cancer cell lines, HEC1A cells and whole blood cell (in vitro)	GLY; GBHs (Roundup and Wipeout)	0–500 µg/mL	GLY and Wipeout reduced cell viability; reduced cell viability was observed in HEC1A exposed to GLY (75–500 µg/mL) and proliferative effects were observed after the exposure to Wipeout (75–250 µg/mL); DNA damages were detected in the HEC1A and MDA-MB-231 cells exposed to GLY and GBHs	Co-formulants and/or GLY impurities were possible contributors to toxicity based on the differential toxicological profiles of GBHs	de Almeida et al. (2018)
Sprague–Dawley rats (in vivo)	GLY; GBH (Glyfonova 450 PLUS)	2.5 and 25 mg/kg bw	Limited effects were detected on bacterial community composition in Sprague–Dawley rats exposed to the GBH and GLY individually	Harmful effects of GLY cannot be excluded in the case of human malnutrition or special diets (e.g., low protein) may cause lower levels of available amino acids in the gut	Nielsen et al. (2018)
Albino rats (in vivo)	GLY; GBH (Roundup)	3.6, 50.4, and 248.4 mg/kg bw GLY equivalent	Altered level of the kidney function biomarker, oxidative stress markers, and membrane-bound enzymes indicating nephrotoxicity (Roundup: 50.4 and 248.4 mg/kg bw GLY equivalent); kidney function was not affected by GLY	The detected nephrotoxicity of Roundup cannot be linked to the AI	Dedeke et al. (2018)
Human mononuclear white blood cells (in vitro)	GLY; GBHs (Roundup Mega, Fozat 480 and Glyfos)	0.17–169 mg/L GLY equivalent	Increased cell death (≥ 42.27 mg/L Roundup Mega and Glyfos and 84.55 mg/L Fozat 480 GLY equivalent) and DNA damage (84.55 mg/L Roundup Mega and Glyfos and 126.8 mg/L Fozat 480 GLY equivalent); geno- and cytotoxicity of GLY were not detected	The different toxicity of the GBHs and GLY can be explained by the high cytotoxicity of the co-formulant or the interaction between the AI and the other components	Nagy et al. (2019)
HepG2, A549, and SH-SY5Y human cell lines (in vitro)	GLY; ethoxylated formulants (e.g., POEA [4130 VR]); mixtures of the AI and the formulants	Assay- and compound-specific concentration range	Inhibitory effect on cell proliferation after the exposure to ethoxylated formulants and their mixtures with GLY; significant toxicity of GLY was not detected	Toxic effects of GBHs can be explained by primarily due to the use of formulants	Hao et al. (2019)
Sprague–Dawley rats (in vivo)	GLY; GBH (Roundup)	5 mg/kg bw GLY equivalent	Altered gut microbiota (especially the phyla *Bacteroidetes* and *Firmicutes*) in the exposed mother rats	GLY alone and in formulation caused altered maternal behavior, neuroplasticity and gut microbiota	Dechartres et al. (2019)
Albino rats (in vivo)	GLY; GBH (Roundup Original)	3.6, 50.4, and 248.4 mg/kg bw GLY equivalent	Severe metabolic disturbance and stress were induced in rats exposed to the GBH; a mild change was observed in the general metabolism of the rats exposed to GLY (54.8 mg/kg bw)	Severe metabolic disturbance and stress were detected in rats exposed to the GBH, but these observations are not connected to the mild effects induced by the AI	Owagboriaye et al. (2019)
Rat Sertoli cell (in vitro)	GLY; GBH (Roundup Full II)	0.01 to 1 g/L	The expression of proteins forming the blood–testis barrier, the lactate production, and the fatty acid oxidation was not affected; decreased transepithelial electrical resistance was detected indicating the establishment of a Sertoli cell junction barrier; delocalization of the signal from membrane to the cytoplasm was induced	GLY and GBHs could alter the function of Sertoli cell (e.g., blood–testis barrier integrity) and thus may compromise the spermatogenesis	Gorga et al. (2020)
Pig semen (in vitro)	GLY; GBH (Roundup Bioflow)	0–360 µg/mL GLY equivalent	Decreased sperm motility, viability, mitochondrial activity, and acrosome integrity after the exposure to GLY (360 µg/mL) and GBH; no effects on sperm DNA integrity	Negative effects were detected on male gametes in the treated groups, but Roundup was more toxic compared to the pure GLY	Nerozzi et al. (2020)
Pig oocyte (in vitro)	GLY; GBH (Roundup Bioflow)	0–360 µg/mL GLY equivalent	Nuclear maturation and embryo cleavage were not affected, oocyte developmental competence was damaged in terms of blastocyst rate and cellularity after the treatments; altered steroidogenesis and increased oocyte ROS levels were observed after the GBH exposure	Co-formulants enhance the toxicity of the GBH and/or are biologically active components from the viewpoint of the side effects	Spinaci et al. (2020)
Human lung A549 cells (in vitro)	GLY IPA; GBH (Roundup); POEA (Witcamine 4130 A)	GLY IPA, Roundup (100 μg/mL GLY equivalent); POEA (35 μg/mL)	Damage of mitochondrial membrane, activation of caspase-9/-3, cleavage of poly (ADP-ribose) polymerase, oxidative DNA damage, DNA single-strand breaks and double-strand breaks were observed in Roundup- and POEA-treated A549 cells; no effects of GLY was observed	The effects of Roundup on the apoptosis and DNA damage of the exposed human A549 cells is connected to the presence of POEA in the GBH	Hao et al. (2020)
Rat (in vivo)	GLY; GBH (Magnum Super II)	2 mg GLY/kg/day	Preimplantation losses were induced in F1 rats; higher 17β-estradiol serum levels and uterine estrogen receptor alpha protein expression; while no effects on progesterone levels and at the transcript level; GLY decreased progesterone receptor mRNA expression	Perinatal exposure to pure GLY and the GBH disrupted critical hormonal and uterine molecular targets during the receptive state; the AI may be the main responsible for the adverse effects	Lorenz et al. (2020)
Human MUTZ-3-derived cells (in vitro)	GLY; GBHs (Roundup Flex and Jablo); POEA	GLY: 500 μM; GBHs (Roundup Flex 0.12%, Jablo 0.008%); POEA: 0.001%	GLY was classified as a non-sensitizer based on in vitro assessment; GBHs and POEA were identified as skin sensitizers	The mixture of POEA and GLY has a similar sensitizing effect as POEA alone, indicating that GLY may not enhance the sensitizing potential when combined with POEA	Lindberg et al. (2020)
Human peripheral blood mononuclear cells (in vitro)	GLY; GLY metabolites (AMPA, methylphosphonic acid); GLY impurities (e.g., N-methylglyphosate)	0.01–5 mM	Altered membrane permeability; induced caspase activity and chromatin condensation; possible induction of apoptosis both via extrinsic and particularly intrinsic pathway	Similar effects on apoptotic parameters were observed after the treatments, but especially at the higher concentration ranges	Kwiatkowska et al. (2020)
Human peripheral blood mononuclear cells (in vitro)	GLY	0.5, 10, and 100 μM	Significant reduction in global DNA methylation level (≥ 0.5 μM), altered expression of genes involved in the regulation of cell cycle and apoptosis	Possible disruption in methylation processes and gene expression was observed, but final metabolic effects were not affected	Woźniak et al. (2020)
Juvenile rats (in vivo)	GLY; GBH (Roundup)	2 and 50 mg/kg/day	Altered blood–testes barrier permeability; induced testicular histological lesions characterized by disorganized seminiferous epithelium, with apparent low cellular adhesion	Continuous exposure to low doses of GLY and GBH affects blood–testes barrier permeability in juvenile rats	Gorga et al. (2021)
Human mononuclear white blood cells (in vitro)	GLY; GBHs (Roundup Mega, Fozat 480, and Glyfos)	0.02–1691 mg/L GLY equivalent	Cytotoxicity of GLY was not detected; cell death was induced after the GBHs exposure (≥ 169 mg/L GLY equivalent); increased frequency of micronucleus formation (GLY: 16.9 mg/L; Glyfos and Fozat 480: ≥ 1.69 mg/L GLY equivalent); genotoxicity of Roundup Mega was indicated (16.9 mg/L GLY equivalent)	The presence of co-formulants in GBHs or the interaction between the AI and co-formulants is responsible for the increased toxicity of GBHs	Nagy et al. (2021)
Sprague–Dawley rats	GLY; GBHs (Roundup Bioflow, i.e., MON 52276)	0.5, 50, and 175 mg/kg bw per day GLY equivalent	Ceca accumulation of shikimic acid and 3-dehydroshikimic acid was induced indicating the inhibition of 5-enolpyruvylshikimate-3-phosphate synthase of the shikimate pathway in the gut microbiome increased the levels of cysteinylglycine, gama-glutamylglutamine, and valylglycine in the cecal microbiome	The shikimate pathway was inhibited in the gut microbiome of the treated rats	Mesnage et al. (2021a)
Human placenta (ex vivo)	GLY; GBH (Roundup GT+)	1 mg/L GLY equivalent	Altered placental permeability of antipyrine, fetal venous flow rate, and the destruction of fetal vessels were observed after the GBH exposure	The fetal–placental circulation and integrity was affected by the GBH according to exposure time; co-formulants and impurities in the GBHs might be responsible for the observed toxicity	Simasotchi et al. (2021)
Pig spermatozoa (in vitro)	GLY; GBH (Roundup Ultra Plus); POEA	6.93–27.74 mg/L GLY equivalent	Increased plasma membrane disorganization, decreased sperm motility, and inhibited phosphorylation pathways in a dose-dependent manner after GBH- and POEA treatments; functional sperm parameters were not affected by GLY	Effects of low Roundup concentrations on pig spermatozoa function are possibly caused by the presence of co-formulants and not the AI GLY	Torres-Badia et al. (2021)
Human peripheral blood mononuclear cells (in vitro)	GLY; AMPA	GLY: 0.5–100 µM; AMPA: 0.5–250 µM	Altered expression of genes involved in the DNA methylation and the modification of histone deacetylation in the exposed cells (≥ 0.5 µM GLY; ≥ 10 µM AMPA)	Altered expression of genes involved in the regulation of transcriptionally inactive chromatin, but GLY increased the gene expression at a lower concentration compared to AMPA	Woźniak et al. (2021)
Cattle rumen content	GLY; GBHs (Durano TF and Roundup LB plus)	0.1 mg/L, 1.0 mg/L, or 10 mg/L GLY equivalent	The shikimate pathway, fermentation parameters (e.g., pH, redox potential, NH_3_-N concentration), microbial diversity or abundance of microbial taxa were not affected	Bacterial communities of the cattle rumen were not affected by GLY and its formulation	Brede et al. (2022)
Sprague–Dawley rats (in vivo)	GLY; GBHs (e.g., Roundup Bio Flow, Roundup ProBio, Roundup PROMAX)	0.5, 50, and 175 mg/kg bw/day GLY equivalent	Increased hepatic steatosis and necrosis were observed after Roundup Bio Flow exposure; altered expression of genes in liver reflecting TP53 activation by DNA damage and circadian rhythm regulation was detected after GLY and Roundup Bio Flow treatments; GLY increased DNA damage formation in liver	Roundup formulations induce more biological changes associated with carcinogenesis than GLY alone	Mesnage et al. (2022a)
Human intestinal epithelial Caco-2 and hepatocyte HepG2 cell lines (in vitro)	GLY; GBH (RangerPro); POEA	Assay- and compound-specific concentration range	RangerPro and POEA were more cytotoxic than GLY alone; cell necrosis was observed in both cell lines exposed to POEA and GBH; GLY and its formulation caused oxidative stress in HepG2 cells; endoplasmic reticulum stress was detected after the exposure to POEA and GBH	The toxicity of the GBH is multifactorial, involving endoplasmic reticulum stress induced by the co-formulant and oxidative stress caused by GLY	Mesnage et al. (2022b)
Mammalian stem cell (in vitro)	GLY; GBHs (e.g., Roundup Bio Flow, Roundup ProBio, Roundup PROMAX)	0.5, 50, and 175 mg/kg bw/day GLY equivalent	Oxidative stress and unfolded protein responses were observed after the exposures to Roundup Bio Flow and Roundup ProBio	Roundup formulations induce more biological changes compared to the individual toxicity of GLY	Mesnage et al. (2022a)
Rat	GLY; GBHs (Roundup Bioflow and RangerPro)	0.5, 5, and 50 mg/kg bw/day GLY equivalent	Fungal and bacterial diversity was affected by GBHs in dose-dependent way, while GLY affected significantly only the bacterial diversity; the taxonomical effect was more pronounced in females	Significant alteration was indicated in caecum microbiome composition of rats exposed to GLY and GBHs	Mesnage et al. (2022c)
Human neuroepithelial stem cells (in vitro)	GLY; GBH (Roundup Transorb)	2.5 mg/L GBH; GLY in equivalent concentration	A more immature neuronal profile (e.g., a shift in differentiation toward glial cells at the expense of mature neurons) was indicated after GBH exposure	Potential long-lasting impairments were detected on the differentiation of human neuroepithelial stem cells exposed to the GBH	Reis et al. (2022)
Rat (in vivo)	GLY; GBH (Roundup Active); AMPA	GLY, GBH: 5 mg/L GLY equivalent; AMPA: 5 mg/L	GLY resulted in a moderate level of glial fibrillary acidic protein without overlapping astrocyte processes, while overlapping was detected after the exposure to GBH and AMPA; higher cell proliferative activity was induced by AMPA and the GBH	Altered immunoreactivity, glial activation, and induction of apoptotic pathways were observed in the rat hypothalamus, modifying the neuroendocrine axis	Duque-Díaz et al. (2022)
Human hepatoma HepG2 cells and human telomerase immortalized human fibroblast cell line (in vitro)	GLY; GBH (Roundup Probio)	GLY: 100, 250, 500 µg/mL; GBH: 110, 220, 440 µg/mL	Higher cytotoxicity was indicated for the GBH compared to the individual toxicity of the AI; oxidative stress response was induced by GLY (250 µg/mL) compared to the GBH	Higher toxicity was proved for the GBH compared to the individual toxicity of the AI GLY	Ferguson et al. (2022)
Neuroectodermal stem cell-like (NE-4C) and osteoblastic cell lines (MC3T3-E1) (in vitro)	GLY; GBH (Roundup Classic); POEA	Assay- and compound-specific concentration range	The order of cytotoxicity and inhibitory potency of the tested compounds has been the following POEA > Roundup Classic ≫ GLY	POEA was the most cytotoxic substance, the presence of the co-formulant in GBHs or the interaction between the AI and co-formulant is responsible for the increased cytotoxicity of the GBH	Oláh et al. (2022)
New Zealand rabbits	GLY; GBH (Roundup)	0.5 mg/kg bw	Disturbances of blood redox equilibrium were observed after the treatments; the mixture of Roundup and endocrine disruptors induced oxidative stress in the liver tissue	The long-term, low-dose exposure to the endocrine disruptor mixture and Roundup caused adverse effects on the liver’s redox status of rabbits	Vardakas et al. (2022)
Human blood cells (in vitro)	GLY; GBHs (Faena, Tackle, and Centella)	0.017, 0.17, 1.69, and 8.46 g/L GLY equivalent	Genetic damage was induced after the exposure to GLY, Faena, and Tackle; increased frequency and tail lengths of some migration groups were caused by GLY and GBHs; Centella decreased migration range and increased frequency of migration groups were detected	Compared to the individual genotoxicity of GLY, higher genotoxicity was indicated for GBHs, due to the presence of co-formulants	Alvarez-Moya and Reynoso-Silva (2023)
Rat (in vivo)	GLY; GBH (Roundup)	Systemic exposure 75 or 150 mg/kg i.p.; intrastriatal exposure: 1, 5, or 10 mM	Significant concentration-dependent increase in dopamine release; systemic exposure to GLY significantly damaged motor control and decreased striatal acetylcholinesterase activity and antioxidant capacity	GLY may have an additive effect on dopamine levels when combined with nomifensine, indicating the need for further research	Costas-Ferreira et al. (2023)
Human TK6 cells (in vitro)	GLY; GLY IPA; GBHs (e.g., Cornerstone Plus, Roundup Custom); AMPA	Assay- and compound-specific concentration range	Genotoxicity or significant cytotoxicity was not detected after the exposure to GLY and AMPA; GBHs were found to be cytotoxic, and some GBHs showed genotoxic activity	The absence of genotoxicity for GLY suggests that the observed toxicity linked to other components presented in the GBHs	Smith-Roe et al. (2023)
Human glioblastoma cell line (A172) (in vitro)	GLY; GBH (Roundup Original DI); AMPA	0.001–50 mg/mL	Time-dependent cytotoxic effects; altered cell cycle and DNA damage; GLY triggered the activation of NLRP3 immunoreactivity, resulting in the recruitment of caspase-1, while AMPA reduced NLRP3 immunocontent, and GBH did not affect this pathway	GLY, GBH, and AMPA may influence cell signaling pathways, leading to oxidative damage and inflammation and providing glioblastoma cells a competitive advantage by enhancing their proliferation and growth	Bianco et al. (2023)
Human hepatocellular carcinoma (HepG2) cell line (in vitro)	GLY; GBH (Roundup Star)	GLY (0–1000 μM); GBH (0–500 μM)	Increased cell proliferation (GLY: 200 μM, GBH: 50 and 100 μM); increased ROS levels (GBH: 25 and 100 μM, GLY: 100 and 200 μM)	Increased expression levels of genes related to the mitogen-activated protein kinase/extracellular signal-regulated kinase signaling pathway were observed after the treatments	Mehtiyev et al. (2023)
Mice (in vivo)	GBH (RoundUp)	0, 50, 250, and 500 mg/kg/day	Hepatocyte structural changes, inflammation, mitochondrial swelling and vacuolization, damaged liver function and aggravated oxidative stress (≥ 250 mg/kg/day)	Hepatotoxic effects of GBH, the induced oxidative stress disrupts energy metabolism, and triggers an inflammatory response in the liver of the exposed mice	Qi et al. (2023)

*GLY* glyphosate, *GLY IPA* GLY-isopropylammonium salt, *GBH* GLY-based formulations, *POEA* a mixture of polyethoxylated tallow amines, *APG* alkyl polyglucosides, *FSH* follicle-stimulating hormone, *IGF-1* insulin-like growth factor 1, *AI* active ingredient, *AMPA* aminomethylphosphonic acid, *bw* bodyweight, *ROS* reactive oxygen species, *i.p*. intraperitoneal injection

## Impacts of glyphosate and its formulations on human health outcomes

Two realities pervade GLY and GBHs human health risk assessments and judgments (Portier et al. 2015, 2016). First, opinions differ over whether GLY and GBH exposures are associated with any adverse health outcomes. The International Agency for Research on Cancer (IARC), operating within the World Health Organization (WHO), classified GLY as “probably carcinogenic to humans” (2A) in March, 2015 (IARC 2015; Székács and Darvas 2018). In general, GBH registrants and their allied scientists and consultants, and most regulators, continue to believe and/or assert that GLY and GBHs pose no meaningful human health risks. A majority of independent scientists conducting GLY and GBHs toxicological and/or epidemiology research often report one or more findings suggestive of such associations. Several recent papers characterize the evidence linking GLY, AMPA, and/or GBH exposures to specific adverse health outcome as “strong” or “compelling,” and especially among those heavily exposed over long periods of time (Mesnage and Antoniou 2018; Zhang et al. 2019; Benbrook et al. 2023; Rana et al. 2023).

Second, the volume and quality of published GLY and GBH studies has exploded in the last decade, triggered in no small part in 2015 by the unexpected IARC classification of GLY and GBHs as “probable” human carcinogens. The commencement soon thereafter of litigation in the US brought by thousands of individuals alleging their applications of Roundup caused or contributed to their non-Hodgkin lymphoma further intensified research interest. Indeed, the substantial increase since 2015 in independent, published research on GLY and GBH health risks is in stark contrast to the near absence of any significant new, registrant-commissioned research since the 2000s on GLY and GBH ADME, toxicity, and health impacts. We believe it is likely that emerging science will soon further elucidate both the mechanisms that can lead to adverse outcomes in the wake of GLY and GBH exposures, as well as the magnitude of such risks among heavy users of GBHs and, in time, the general public.

The US Environmental Protection Agency (US EPA), the European Food Safety Authority (EFSA), and other regulatory authorities consider dietary exposures to active substance GLY to be “probably not carcinogenic to humans” (EFSA 2015a; Székács and Darvas 2018; US EPA 2018). The most recent EFSA GLY risk assessment was published by EFSA on July 26, 2023 (EFSA et al. 2023; Klátyik et al. 2024). The approval of GLY registrations in the EU was approved by the European Commission in late 2023 for 10 years (European Commission 2023). However, no regulatory agency worldwide has in its possession the data needed to carry out an accurate GBH applicator, dermal exposure risk assessment. Moreover, regulators have chosen not to take the steps needed to generate or obtain essential worker-exposure data in light of the generally low acute toxicity of GBHs. In addition, it is important to consider the fact that by law and regulation, the primary focus of regulators is minimizing general population risks stemming from GLY residues in the diet. In this regard, in its 2023 GLY renewal decision, the EU Commission and EFSA did impose meaningful restrictions on the GBH uses most responsible for residues in food. They did so by banning preharvest crop dessication applications in Europe, although the steps needed to prevent GLY residues from continuing to enter the EU food supply via imported grains, beans, and certain oilseeds were not taken. Hence, GLY residues stemming from pre-harvest GBH applications on certain crops from some countries will remain a concern (e.g., in particular, the US).

Our review focuses on studies published since the EU Commission Directive 2010/77/EU. Systematic searches were carried out within the Web of Science, Scopus, Science Direct, and other scientific databases. In addition, selected references in published reports were assessed. This review relies predominately on studies deemed relevant to the evaluation and/or quantification of potential environmental and toxic effects of GLY and GBHs. Subsequent sections are organized by mode of action of GLY, GBHs, and GBH co-formulants.

## Modes of herbicidal action and side effects of glyphosate and its formulations

**Fig. 1:**
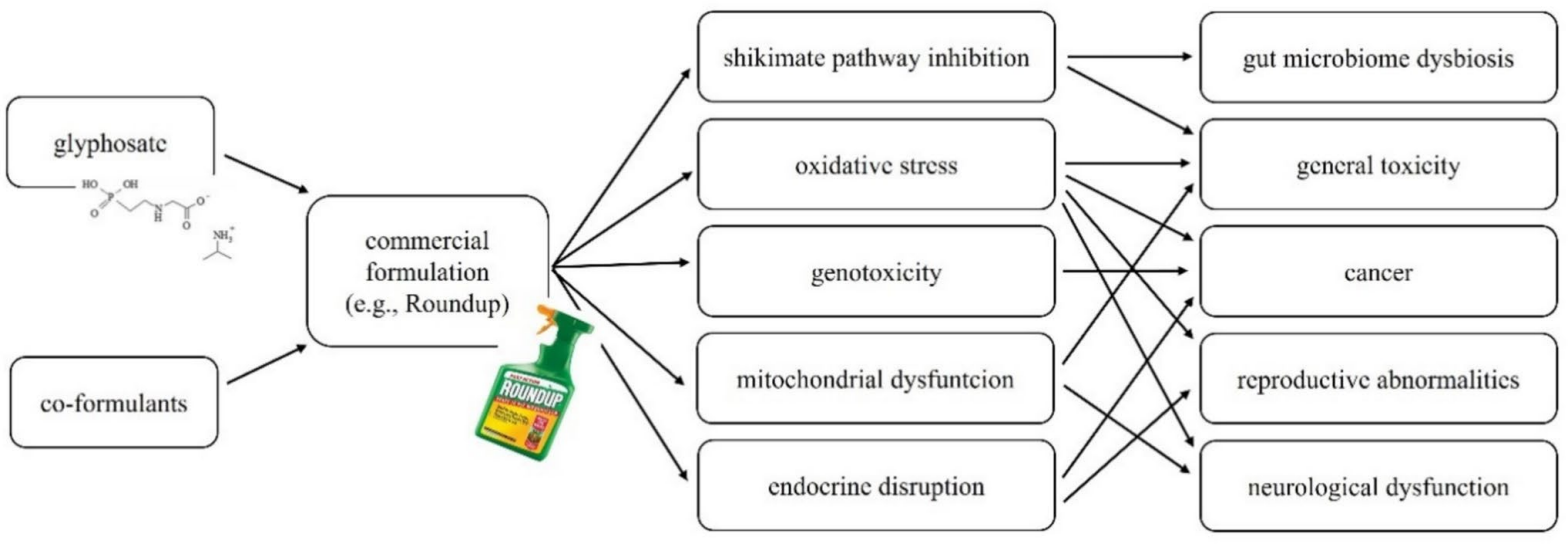
Modes of action leading to identified side effects of glyphosate, GBHs, and co-formulants, and possible corresponding physiological disorders

GLY is used in herbicide formulations for plant protection purposes. In GBHs, the various salts of GLY (e.g., GLY-isopropylammonium salt [GLY-IPA]) are incorporated as AIs to increase water solubility (Defarge et al. 2016; Travlos et al. 2017). Besides the AI, co-formulants are also added in commercial GBHs. The primary purpose of co-formulants is to increase the effectiveness of the GLY in the formulation by enhancing adherence to weed leaf tissues, even in the event of rain, and hastening the movement of GLY through the epidermis of weed leaf tissues (Foy 1987; Mesnage and Antoniou 2018). In addition, the co-formulants in GBHs facilitate the penetration of GLY into plant cells (Defarge et al. 2018). Co-formulants have long been considered as inactive components in terms of weed control. However, several studies elucidate the ways in which co-formulants actually enhance GBH potency. In addition, and less welcomed, co-formulants also increase the toxicity of formulated GBHs compared to GLY alone (Mesnage et al. 2013a, 2019, 2022a; Maderthaner et al. 2020). This is especially the case with POEA surfactants and genotoxicity (Székács 2017; Mesnage et al. 2019; Langrand et al. 2020). Conversely in a few cases, GLY alone has proven more toxic than GBHs (Mesnage et al. 2017). For example, reduced biomass and survival were demonstrated in earthworms exposed to GLY-IPA compared to the control, while significant effects were not detected in earthworms exposed to GBHs (Pochron et al. 2020). Similar formulant-related toxicities of GBHs Liquidator (obtained from Poland), as well as Glider and Tornado (obtained from Russia) detected in an oxidoreductase/luciferase enzymatic inhibitory assay were also reported (Esimbekova et al. 2025). The main herbicidal action, and identified side effects of GLY and its formulations, impose risks via a wide range of biochemical/physiological modes of action (Fig. 1).

Since the EU ban of POEA co-formulants in GBHs, alkyl polyglucosides and quaternary ammonium (e.g., alkyl dimethyl betaine) co-formulants are generally incorporated in GBH formulations (e.g., Touchdown Quattro, Agpro Glyphosate 510, Roundup ProVantage, Roundup Biactive). Alkyl amines and its derivates (e.g., alkyl ether amines) are also widely used in GBHs as adjuvants (e.g., Monsanto Amenity Glyphosate XL, Monsanto Amenity Glyphosate 360, Glyfos ProActive) in addition to other additives (e.g., water, stabilizers, solvents, and safeners). According to current EU legislation, all AIs have to undergo an authorization process at the EU level (European Parliament and the Council 2009). Member states are responsible for the assessment of GBH-specific uses, exposures, and applicator and bystander risks, but do so with minimal new data beyond the studies accessible to EFSA on parent compound GLY. This is a major shortcoming given that an already large and growing body of published in vitro and in vivo studies have documented substantially greater adverse impacts stemming from exposures to the GLY in GBH mixtures compared to the same amount of GLY alone (Defarge et al. 2018; Vanlaeys et al. 2018; Mesnage et al. 2019, 2022a, 2022b; Maderthaner et al. 2020). However, the ability of independent scientists to elucidate the impacts of different co-formulants in commercial GBHs is hindered by laws and regulations that allow GBH manufacturers to claim the identity and concentration of GBH co-formulants as confidential business information. Scientists can study the collective impacts of the co-formulants in various GBHs but cannot know for sure which specific chemicals are responsible for heightened toxicity. This is why nearly every published paper calling for the reform of pesticide law and policy calls for changes necessary to end Confidential Business Information (CBI) protection for pesticide co-formulants (Mesnage et al. 2019; Fisher et al. 2023; Straw 2024).

### Modes of the herbicidal action in plants

During a GBH application, a small part is lost to the atmosphere and most of the formulated product lands on weed or GM crop plant leaf tissues, or on the soil surface. Thus, the aerial loss does not vary by the weediness of the crop field, while GLY movement onto soil does: the heavier the weed infestation and/or GM crop plants, the greater the portion of GBH applied that lands on weeds or crop plants. GLY that penetrates the weed epidermis is transported by phloem down into roots, where it is exuded into the soil (Helander et al. 2012; Hébert et al. 2019). GLY exerts its herbicidal effects by the inhibition of the 5-enoylpyruvylshikimate-3-phosphate synthase (EPSPS) enzyme within the shikimate pathway. GLY applications result in premature aging, necrotic alterations, and lethal effects on plants. Since the shikimate pathway is found in all plants, GLY acts as a broad-spectrum, non-selective herbicide. GLY is referred to in various studies as a mild, moderate, and strong chelating agent (often pH dependent). Chelation is an important physicochemical property of chemicals, affecting their reactivity and ability to bind with other molecules. The weakly negative charge and structure of GLY molecules depends on pH and the specific chemical form of the AI (e.g., GLY-IPA) in a given GBH (Henderson et al. 2010). The inhibition of the EPSPS enzyme, which catalyzes the conversion of phosphoenolpyruvic acid (PEP) and 3-phosphoshikimic acid to 5-enolpyruvyl-3-phosphoshikimic acid, occurs due to competition between PEP and GLY for binding to the enzyme’s active site. Consequently, the reactivity of GLY, and its ability to bind with other molecules, is essential to its effectiveness as an herbicide AI. Due to the inhibited formation of the central intermediate of the biosynthesis of aromatic amino acids, the synthesis of secondary metabolite is also reduced (Nielsen et al. 2021).

GLY is a zwitterion with three acidic protons, enabling it to act as a tridentate chelating agent for divalent and trivalent metals, forming 1:1 or 1:2 complexes (Vicini et al. 2019). GLY treatments may reduce mineral availability in plants by restricting soil uptake, or by impairing phloem transport of minerals (Duke et al. 2012). Greenhouse experiments on soybean plants suggest that GLY may disrupt the uptake and redistribution of certain divalent cationic nutrients (e.g., Ca, Mg, Fe, and Mn), most likely by sequestering and immobilizing them (Cakmak et al. 2009). The disruption of mineral absorption in the digestive tract of mammals (e.g., Co in ruminants) due to chelation of ingested GLY residues has also been reported (Motekaitis and Martell 1985; Harris et al. 2012). In response to claims regarding the binding of GLY to essential mineral micronutrients in plant phloem or the human gastrointestinal tract, Vicini et al*.* highlighted that there are more reactive molecules and minerals in these environments compared to GLY (Vicini et al. 2019). However, Vicini et al*.* do not discuss the propensity of GLY to bind with calcium in bone tissue but do address GLY binding to calcium in plants or soil matrices. GLY can interact with calcium in bone and can be immobilized in bone by chelation, resulting in longer retention of GLY in the body (Benbrook 2025). While the chelating properties of GLY are well known, the possible environmental and toxicological risks arising from chelation and immobilization in mammals have not been adequately considered during the re-evaluation and regulatory risk assessment of GLY (EFSA 2015a, 2015b; Mertens et al. 2018).

The shikimate pathway is also found in most fungi and some bacteria. With the exception of organisms in the mammalian microbiome and human skin, the shikimate pathway is absent in animals (Boocock and Coggins 1983; Herrmann and Weaver 1999). Consequently, fungal and bacterial populations can be affected by the increased use of GBHs (Tohge et al. 2013; Klátyik et al. 2023; Li et al. 2025). Moreover, secondary indirect effects of GLY were indicated by alterations in the endophytic and rhizosphere microbiome of plants (Berg et al. 2014; van Bruggen et al. 2021). Disruption of microbiomes can result in decreased antimicrobial production (van Bruggen et al. 2015). The increased exudation of amino acids and carbohydrates via the roots of GLY-exposed plants may attract and nourish pathogens, triggering spikes in population levels and disease pressure (Kremer and Means 2009). Furthermore, impaired plant defense processes can promote the entry of pathogens into plants (Duke 2018; Hammerschmidt 2018; Fuchs et al. 2021). In the obligate holoparasitic weed *Phelipanche aegyptiaca*, inhibited translocation of phloem-mobile solutes was observed from the host plant to the parasite. Moreover, disruption was observed in the metabolism of major sugars (Shilo et al. 2017). The side effects of GLY and GBHs exhibited a wide range of additional biochemical and physiological alterations. These include adverse effects on photosynthesis (e.g., inhibited chlorophyll biosynthesis and altered photochemical reactions) (Vivancos et al. 2011; Zobiole et al. 2012; Gomes et al. 2017a), carbon, and nitrogen metabolism (Zobiole et al. 2010; Ding et al. 2011). Moreover, enzyme activities (e.g., catalase and ascorbate peroxidase), and the level of phytohormones, may be also affected by GBHs (Miteva et al. 2010; Mkandawire et al. 2014).

### Effects via the inhibition of the shikimate pathway

The shikimate pathway can be found not only in plants but also in fungi, bacteria, and protozoa, rendering many microbial taxa sensitive to the effects of GLY (Duke 2018). Although the sensitivity of different microorganisms depends heavily on the class of EPSPS and variations in DNA sequences coding for the EPSPS enzyme (Mesnage and Antoniou 2020; Leino et al. 2021; Rainio et al. 2021). The intensive and long-term use of GLY results in the selection of bacterial and fungal strains with low sensitivity to GLY via different resistance mechanisms. These range from the low permeability of the cell wall, to altered EPSPS binding sites, and active removal from the cell (Staub et al. 2012; Liu et al. 2013; van Bruggen et al. 2018; Massot et al. 2019). Moreover, long-term GBH use may lead to cross-resistance against antibiotics for different bacteria, e.g., *Escherichia coli*, *Salmonella* spp., and other environmental bacteria (Kurenbach et al. 2015, 2018; Wicaksono et al. 2021). Furthermore, in clinical and environmental *Pseudomonas aeruginosa* isolates, decreased susceptibility to the potent carbapenem-type antibiotic imipenem was demonstrated in vitro after exposure to 0.5% GBHs. Based on the determined fractional inhibitory concentration indexes, GLY acid and the GBH formulation led to a potent antagonistic effect in all of the investigated *P. aeruginosa* strains (Háhn et al. 2022). Hence, we conclude that GLY may increase antibiotic resistance of some pathogenic bacteria, not through gene exchange among shared molecular targets, but by mechanisms including efflux pump activation and/or promotion of mutations associated with emergence of resistant phenotypes. These effects are not exclusive to GLY, as similar impacts can be caused by other herbicides. GLY may also enhance genetic exchange of antibiotic-resistance elements through effects on membrane permeability. However, clear evidence of GLY and/or GBH mutagenicity in bacteria is limited, in part because bacteria lack mitochondria, the primary target of GLY-induced oxidative stress (Killham and Prosser 2007; Gomes and Juneau 2016; Strilbyska et al. 2022). This is one of the reasons why more long-term field studies are needed to confirm GLY’s impact on antibiotic resistance genes in soil (Bearson et al. 2024).

Differences in the sensitivity of various microorganisms to GLY can result in altered microbial composition in a variety of habitats including soil, surface waters, plant surfaces, and animal intestinal tracts (van Bruggen et al. 2018). Soil microorganisms play crucial roles in soil ecosystems and energy flows. The soil-specific effects of GLY on bacterial communities include altered density, diversity, biosynthesis and cell growth pathways, metabolism, nitrogen metabolism, and xenobiotics biodegradation pathways (Klátyik et al. 2023; Li et al. 2025). Although the results of the reported studies are sometimes contradictory (Klátyik et al. 2023), the effects of GLY and GBHs on the nitrogen cycle of soil microbial biomass must be taken seriously in the design and verification of “climate-smart” agriculture, especially if and as substantial subsidies are offered for adoption of such systems.

GLY is known to interact with different bacteria, including gut microbiota. The inhibitory effect of GLY on EPSPS affects mainly beneficial bacteria of intestinal microbiota, while *Salmonella* and *Clostridium* spp. strains proved to be resistant to GLY-induced dysbiosis in the gut, including imbalances in the composition of beneficial and pathogenic microorganisms (Rueda-Ruzafa et al. 2019). The disruption of the microbiota by GBHs can trigger or augment effects on human health (e.g., increased risk of autoimmune or cardiovascular disease) (Hu et al. 2021; Zhu et al. 2025). Disruptions were observed in the gut microbiome of earthworms (*Alma millsoni, Eudrilus eugeniae*, and *Libyodrilus violaceus*) exposed to a GBH (Roundup Alphée, 8.3 kg AI/ha). A significant shift occurred in the composition of bacterial populations, including increased abundance of *Enterobacter, Pantoea*, and *Pseudomonas* compared to the control (Owagboriaye et al. 2021). GLY and AMPA caused damage to physiological homeostasis resulting in impairments in the digestive gland microbiota, as well as the spreading of opportunistic pathogens such as *Vibrio* spp. (Iori et al. 2020).

Disruption of gut bacteria makes bees more vulnerable to biotic stresses, causing different health effects and reduced resistance to pathogens. Such impacts can lead to the weakening or complete loss of bee colonies. The reduction of dominant and beneficial gut microbiota species was detected after GLY treatment (5–10 mg/L). In addition, concentration-dependent effects were observed on the abundance of beneficial bacteria in the honey bee gut exposed to Roundup. GLY exposure (0.017, 0.17, or 1.7 g/L for 5 days) decreased the expression of antimicrobial peptides (apidaecin, defensin, and hymenoptaecin) and melanization in honey bees. The decreased abundance of beneficial gut bacteria caused immune dysregulation in bees in response to GLY treatment (Motta et al. 2018, 2020, 2022). Moreover, GLY (0.25 g/L in sugar syrup) itself, but not its metabolite AMPA, induced significant changes in honeybee gut microbiota (Blot et al. 2019). In addition, increased susceptibility to infection was observed in insects as a result of GLY-induced melanin inhibition, immune impairment, and altered composition of the microbiota (Smith et al. 2021).

A growing number of studies demonstrate risks to animals due to inhibition of shikimate metabolic pathways affecting microbes. Dietary exposure to GLY can influence the functional condition of the intestinal microbiome (Laptev et al. 2023), and blood parameters in poultry (e.g., white blood cell and phagocytic counts) (Yildirim et al. 2024). The observed functional differences in the microbiome can lead to decreased diversity and disruption of cell signaling and may reduce the efficiency of digestive processes and energy metabolism (Laptev et al. 2023). GLY can affect the human microbiome, as more than 50% of its microorganisms are sensitive to the effects of GLY. However, additional empirical studies are needed to identify the possible effects of GLY on the human microbiome (Nielsen et al. 2021; Mesnage et al. 2022c; Puigbó et al. 2022).

### Neurological effects

GLY can result in imbalances in the composition of beneficial and pathogenic microorganisms in the nervous system. The overgrowth of bacteria, e.g., Clostridia, can bring about a high level of noxious metabolites in the brain, thereby contributing to the emergence of neurological abnormalities (Rueda-Ruzafa et al. 2019). Recently, the possible neurotoxic effects of GBHs have garnered increasing attention. GBH triggered glutamatergic excitotoxicity in the brain is a potential risk factor for the development of neurodegenerative disorders, including Parkinson’s disease, autism, and Alzheimer’s disease (Eriguchi et al. 2019; Madani and Carpenter 2022; Limberger et al. 2020; Solomon et al. 2024). However, additional investigations are needed to understand the possible mechanisms and clinical significance of GLY/GBH exposures and neurological conditions and decline (Hsiao et al. 2023). However, the scientific evidence remains inconclusive, it is enough to suggest a biologically probable link between GLY exposure and neurotoxic effects (e.g., nigrostriatal cell death), and hence a potential risk for Parkinson’s disease (Bloem and Boonstra 2023).

Other significant adverse effects of GBHs have been demonstrated on the structure and function of the brain. Due to the known importance of glycosylation in disease progression, the glycome profile was investigated in the hippocampus and prefrontal cortex of juvenile rats after the chronic oral exposure to a GBH. Gender-specific responses were observed compared to the control groups (Solomon et al. 2024). GLY and its formulations induced variable neurotoxic effects, while exposure to GLY at the early developmental stages can affect normal cell development by the deregulation of signaling pathways. Such impacts can result in alterations in neuronal growth, differentiation, and myelination (Costas-Ferreira et al. 2022). In addition, neurophysiological, biochemical, and developmental cerebral deviations can arise, such as altered levels of monoaminergic neurotransmitters and neurotransmission (Martínez et al. 2018) or impaired neurofunctions (Chávez-Reyes et al. 2025). Due to the association of GLY with neurodegenerative diseases, its main metabolite AMPA has been suggested as a potential marker of neurodegenerative diseases (Newell et al. 2025).

Oxidative stress, as well as glutamatergic excitotoxicity, neuroinflammation, deformities, and mitochondrial dysfunction, can be induced (El-Shenawy 2009; Paganelli et al. 2010; Cattani et al. 2017; Winstone et al. 2022), resulting in neuronal death via necrosis, apoptosis, or autophagy. In addition, behavioral and motor disorders can arise (Costas-Ferreira et al. 2022), possibly related to GLY-induced disruption to the gut–brain axis (Rueda-Ruzafa et al. 2019). After exposure to a GBH (400 mg/kg), altered arachidonic acid metabolism was assumed in the brains of adult male Wistar rats since changes were observed in the cerebrospinal fluid arachidonic acid levels. Serum urea content was not affected, suggesting the normal function of the urea cycle. Significantly higher ornithine levels were detected in the treated groups compared to the control brain cells, suggesting a possible imbalance in excitatory amino acids and disruptions in arginine downstream metabolism resulting from GBH exposure may contribute to GBH-induced neurotoxicity (Limberger et al. 2020).

Neurochemical changes were detected in rat offspring after subchronic exposure to a GBH (e.g., glutamatergic overactivity and oxidative damage in the hippocampus), where the observed alterations were associated with depressive behavior in adults (Cattani et al. 2017). Based on a retrospective analysis of patients poisoning with GLY-surfactant herbicides, psychiatric comorbidities including mental disorders (71.6%) and depression (48.6%) were widespread, while the mortality rate was 5% (Liu et al. 2024). A positive correlation was detected between the use of GBHs and autism spectrum disorder, potentially resulting from the overproduction of metabolites by *Clostridium* species, which can lead to an excess of dopamine and its metabolites. The production of reactive oxygen species (ROS) via dopamine quinone species results in oxidative stress and mitochondrial dysfunction (Shaw 2017). After exposure to GLY, neurodevelopmental disorders were demonstrated in rodents (Yu et al. 2018). However, most studies and observed alterations connected to GLY concentrations in the brain vary widely, and the role of the GBH surfactants in neurotoxicity is unclear. Several studies have also indicated a potential link between abnormalities in neurodevelopment and exposure to GBHs in *Caenorhabditis elegans* (McVey et al. 2016; Naraine et al. 2022). In addition, GLY neurotoxicity was observed in *Rhinella arenarum* (Lajmanovich et al. 2015).

### Effects via the induction of oxidative stress

Dozens of high-quality studies have demonstrated that GLY-induced oxidative stress can trigger imbalances between the production of ROS and the induction of antioxidant defense systems (Rana et al. 2023), can cause cytotoxicity to human dermal fibroblasts (Batista et al. 2025), or can damage the antioxidant mechanism by inhibiting the glycolytic pathway resulting in decreases in respiratory and metabolic indexes (Jiang et al. 2025), and can sometimes lead to the development of chronic and degenerative diseases (e.g., kidney and liver disorders, cardiovascular, neurodegenerative, and respiratory diseases) (Kronberg et al. 2021). Oxidative stress can be detected by the measurement of various biomarkers, e.g., lipid peroxidation, protein carbonyl content, enzyme activities, level of glutathione, and the concentration of malondialdehyde (Webster and Santos 2015; Hackenberger et al. 2018; Dumitru et al. 2019; Hernández-García and Martínez-Jerónimó 2020). GBH induces oxidative stress that disrupts energy metabolism and triggers an inflammatory response in the liver of mice exposed to GBH (≥ 250 mg/kg/day) for 30 days (Qi et al. 2023). In addition, childhood exposure to GLY and AMPA may elevate the risk of liver and cardiometabolic disorders in early adulthood, potentially contributing to the development of more severe diseases later in life (Eskenazi et al. 2023).

GBHs can also mediate the production of free radicals as indicated by increased levels of lipid peroxidation, thiobarbituric acid reactive species, hydrogen peroxide, and the carbonylation of proteins. However increased glutathione-S-transferase activity can indicate a compensatory response against the toxic effects of xenobiotics (Modesto and Martinez 2010; Cattaneo et al. 2011; de Menezes et al. 2011; Xu et al. 2017; Liu et al. 2018). Significantly higher superoxide dismutase activity was detected in human mononuclear white blood cells exposed to GBHs or adjuvants, compared to cells treated with GLY (Makame et al. 2023). Observed effects highlight the important role of co-formulants present in GBH-induced of oxidative stress (Makame et al. 2023). Roundup exposure over 24 h increased levels of antioxidants neutralizing peroxyl radicals and upregulating antioxidant defense system gene expression in *Drosophila melanogaster*. In addition, early activation of the antioxidant defense system was reported in exposed flies (de Aguiar et al. 2016). The toxic mechanisms of GLY in terms of oxidative stress altered signaling pathways and antioxidant status, as well as the regulation of oxidative stress by the exogenous substances (Wang et al. 2022). The production of ROS by GBHs or GLY can affect negatively various non-target organisms due to the inhibitory effects on photosynthesis (de Menezes et al. 2011); Gomes and Juneau 2016) and cholinesterase activity (Braz-Mota et al. 2015). It can also cause cell death (Sulukan et al. 2017; Lanzarin et al. 2021), DNA damages (Nwani et al. 2013; Braz-Mota et al. 2015; Cao et al. 2022), alterations of the stacking pattern of thylakoids in algae cells (Iummato et al. 2019), and hormonal alterations (Liu et al. 2022).

### Cellular effects

GLY is often thought of as a low-risk compound in terms of human health because of the absence of the shikimic acid pathway in mammals. Still, several studies have demonstrated that GLY can provoke cellular alterations known to be associated with the etiology of blood cancers, kidney damage, and neurological diseases (Roulland et al. 2004; Agopian et al. 2009; Schinasi and Leon 2014; Suarez-Larios et al. 2017; van Bruggen et al. 2018; Rueda-Ruzafa et al. 2019; Hu et al. 2025; Khacha-ananda et al. 2025; Benbrook 2025). The IARC Working Group that evaluated GLY and GBH for oncogenic potential identified “strong evidence” of oxidative stress and direct damage to DNA that supported its “probably carcinogenic” classification decision (IARC 2015). The US EPA’s Office of Research and Development (ORD) was asked by the agency’s administrator to review the scientific basis underlying conflicting Office of Pesticide Programs (OPP) of the US EPA and the IARC in the term of GLY and GBH cancer classification decisions. The ORD team concluded that OPP’s “not likely” classification decision would be incompatible with the US EPA cancer risk assessment guidelines if there is ***any evidence*** pointing to GLY/GBH genotoxic or mutagenic potential. Furthermore, the European Chemicals Agency (ECHA) has acknowledged the induction transient DNA strand breaks by GLY in both in vitro and in vivo testing (ECHA 2022).

Several studies have confirmed that GLY and its formulations induce DNA damage in rodents and human cell lines after exposures regarded as roughly equal to acceptable daily intakes in the EU (0.5 μg/mL or ppm). An increased number of micronucleus and chromosomal aberrations were observed in human lymphocytes, as well as stimulation of cell proliferation following GLY exposure (Guyton et al. 2015; Kašuba et al. 2017; Santovito et al. 2018; Nagy et al. 2021). A dose-dependent decrease in T-lymphocyte proliferation was observed following GLY exposure (10–10,000 μg/L), attributed to the upregulation of genes associated with acute-phase inflammation and the suppression of the T-lymphocyte proliferation pathway (De Maria et al. 2024). The potential genotoxic effects of GLY-IPA, Roundup Classic, and POEA were observed in the Comet assay performed on NE-4C and MC3T3-E1 cell lines (Oláh et al. 2022). Furthermore, the presence of surfactants such as POEA promotes the penetration of GLY into plant cells, albeit presenting potential cytotoxic effects (Defarge et al. 2018). The genotoxicity of GLY, and especially its formulations (e.g., Glyfos, Roundup, Glyphogan), were indicated in a wide range of different animal and plant organisms by DNA fragmentation effects and lipid peroxidation (Ghisi et al. 2016; Barbosa et al. 2017), and genotoxic effects such as micronucleus and nuclear abnormalities in blood, liver, and gill cells of fish (*Piaractus mesopotamicus*) (Leveroni et al. 2017). Moreover, mutagenic and genotoxic effects were detected in gill erythrocyte cells in exposed *Poecilia reticulata* (de Souza Filho et al. 2013), amphibians, and plant (Lajmanovich et al. 2011; Alvarez-Moya et al. 2014; Nardemir et al. 2015; Rissoli et al. 2016).

Cytotoxicity of GLY and its formulations has also been demonstrated by mitochondrial functions, cell viability, cell proliferation, the release of lactate dehydrogenase, lysosomal activity, and membrane integrity in several cell types (e.g., human embryonic and placental cells), and test organisms (e.g., fish) (Koller et al. 2012; Mesnage et al. 2013a, 2013b; Defarge et al. 2016; Qin et al. 2017; Sulukan et al. 2017; Cestonaro et al. 2025). However, cytotoxic effects were not detected after the individual exposure to GLY up to 0.17 mg/mL (Song et al. 2012; Kim et al. 2013). Similarly, GLY alone did not significantly affect the viability of human mononuclear white blood cells. However, GBHs and the tested co-formulants (POEA and alkyl dimethyl betaine) induced cytotoxic effects (from 16.91 mg/L GLY equivalent concentration) (Makame et al. 2023). Based on the results of cytotoxicity tests using human intestinal epithelial Caco-2 and hepatocyte HepG2 cell lines, higher toxicity was determined after exposure to RangerPro and POEA, compared to GLY alone. The tested GBHs and co-formulant, but not GLY alone, caused cell necrosis in the tested cell lines. In addition, the induction of oxidative stress was only observed after the exposure to GLY and RangerPro in HepG2 cells (Mesnage et al. 2022b).

Roundup Classic significantly reduced the viability of neuroectodermal NE-4C cells, while POEA inhibited cellular metabolism. According to the results of MTT (3-(4,5-dimethylthiazol-2-yl)-2,5-diphenyl tetrazolium bromide) assays performed on NE-4C and osteoblastic MC3T3-E1 cells, and the order of cytotoxicity was as follows: GLY-IPA << Roundup Classic < POEA. Moreover, a significantly higher level of apoptotic cells was observed in cells exposed to POEA compared to GLY-IPA and Roundup Classic. During the assessment of the cell proliferation cycle, a reduced ratio of cells was demonstrated for all tested compounds after 24 h in the beginning DNA replicating (S) phase. A more significant reduction was observed in cell ratio after 48 h, and in the growth (G_0_/G_1_) phase, an increased ratio of cells was determined compared to the control (Székács et al. 2014, 2016; Oláh et al. 2022). The higher apoptosis-inducing potential of POEA was observed in cell toxicity measurements performed by an optical biosensor and compared to GLY-treated cells at equivalent concentrations (Farkas et al. 2018). A time-dependent reduction was observed in the impacted cell area, as well as increased thickness of NE-4C cells revealed by holographic microscopy after GLY exposure. The exposure to a 0.01% dilution of the formulated Roundup Classic herbicide product (corresponding to 0.0042% and 0.0016% of GLY and POEA concentrations, and being 20-fold below the 2% dilution used in agricultural applications) resulted in a rapid decrease in average cell area due to the extensive cell death (Székács et al. 2014).

In human JEG3 placenta choriocarcinoma cells, comparable cytotoxic effects of GBHs and their formulating agents were demonstrated, while GLY was not cytotoxic below the agricultural dilution of 1% of the formulated GBHs tested (corresponding to 3.6–5.4 g/L GLY concentrations in the dilutions depending on the actual formulation) (Defarge et al. 2016). Furthermore, Roundup Classic and POEA resulted in increased paracellular integrity of IPEC-J2 cells after a 2 h exposure (Pászti-Gere et al. 2015, 2016). The effects of GLY on cellular interactions via Arg-Gly-Asp (RGD)-dependent integrins were detected, while the total inhibition of αvβ3 binding to RGD was caused by GLY and its primary metabolite (AMPA), as well as on α5β1 binding to RGD for acetylglycine (Székács et al. 2018; Gémes et al. 2022). The integrin-inhibitory effect of GLY is structure-specific and was also observed for AMPA, but not for the other investigated structural analogs of GLY (e.g., *N*-acetylglyphosate, glycine, and acetylglycine). GLY and AMPA primarily target the αVβ3 integrin with inhibitory effects on cell adhesion, differentiation, and other vital processes. This disruption can adversely affect cell organization and apoptotic behavior, potentially leading to malignant consequences due to an imbalance in programmed cell death (Gémes et al. 2022).

A growing divergence in regulatory approaches to GLY has emerged in recent years. For instance, the European Commission renewed GLY’s authorization in 2023 for 10 years but included restrictions on preharvest uses in light of concerns over dietary residue levels and the need for certain measures to protect non-target organisms. In contrast, the US EPA continues to endorse GLY without these precautionary measures. Such discrepancies may stem from divergent interpretations of the available toxicological data – most of which focus on the isolated AI GLY, rather than the formulated products that contain surfactants and other “inert” ingredients. In 2015, IARC classified GLY as “probably carcinogenic to humans” (Group 2A) (IARC 2015; Székács and Darvas 2018). According to CLP Regulation (1272/2008 EC) of the EU (European Parliament and the Council 2008), to classify a substance as carcinogenic, at least two positive studies are required. In the case of GLY, the Health and Environment Alliance analyzed 11 animal bioassays provided by pesticide companies as part of the EU renewal dossier, with statistically significant tumors identified in 10 of these studies. Such data presumably support the IARC classification of GLY as a “probable carcinogen” (Lyssimachou and Clausing 2022). Furthermore, several studies and reviews underlined that GLY and its formulations are linked to non-Hodgkin’s lymphoma and the risk of multiple myeloma based on animal experiments. In light of the positive results of rodent bioassays, dozens of GLY and GBH genotoxicity assays, and limited evidence in epidemiology studies, the US EPA and EFSA came to a conclusion that GBHs pose no oncogenic potential among heavily exposed applicators is hard to square with US EPA’s cancer risk classification scheme (Rueda-Ruzafa et al. 2019; Zhang et al. 2019; Weisenburger 2021; de Roos et al. 2022; Benbrook et al. 2023; Rana et al. 2023). However, EFSA and the Committee for Risk Assessment of ECHA classified active agent GLY as likely not carcinogenic via dietary exposure (ECHA 2022). A recent expert report by the French National Institute of Health and Medical Research, Inserm, found a moderate presumption of a link between GBH exposures and increased risk of non-Hodgkin’s lymphoma (Inserm 2022). Yet, other reviews conducted by registrants and/or their surrogates have insisted for decades that there is no causal correlation between GBH use and exposure, and the risk of cancer (Acquavella et al. 2016; Chang and Delzell 2016; Williams et al. 2016). The lack of an adequate number of well-designed cancer bioassays including treatment groups administered GBHs, as well as epidemiology studies among heavily exposed applicators and GLY/GBH manufacturing plants, is wildly recognized as significant data gaps.

### Reproductive and endocrine-disrupting effects

Several studies confirm the reproductive toxicity of GLY and its formulations among non-target organisms, and particularly aquatic invertebrates. The documented adverse impacts include reduced egg production and morphological changes in reproductive organs, tissues, and cells. These effects have induced changes in uterine, testicular, and epididymal tissues, in addition to Sertoli and Leydig cell abnormalities. Embryotoxic malformations have been reported, as well as declining sperm quality (e.g., motility and viability), morphological abnormalities in sperm, and other endocrine-disrupting effects (Romano et al. 2010; Clair et al. 2012; Williams et al. 2012; Abarikwu et al. 2015; Dai et al. 2016; Bridi et al. 2017; Guerrero Schimpf et al. 2017; Sulukan et al. 2017). Effects on gametogenesis and reproductive dysfunction along with dysregulation of various biotransformation proteins have been reported on fish (*Danio rerio*) (Webster et al. 2014; Sulukan et al. 2017; Liu et al. 2022; Morozov and Yurchenko 2025) and rat females and males (Owagboriaye et al. 2017; Hamdaoui et al. 2018) after the exposure to the tested GBH and GLY alone, in addition to effects (e.g., alterations in endometrial decidualization) on adult female rats after the exposure of neonates (Ingaramo et al. 2016). However, the adverse effects on sperm quality are not attributed to pure GLY exposure in rodents based on a recent meta-analysis (Cai et al. 2017).

Observed teratogenic effects and malformations in vertebrates upon exposure to GLY can arise from the consequence of the inhibition of the retinoic acid signaling pathway (Paganelli et al. 2010; Carrasco 2013) and may result in birth defects (Guerrero Schimpf et al. 2017). The toxic effect of GBH on spermatozoa is possibly mediated by the induction of oxidative stress and mitochondrial damage (Lopes et al. 2014). GLY (360 μg/mL) caused a significant reduction in sperm viability, motility, mitochondrial activity, and acrosome integrity in treated pigs. In contrast to the GLY, Roundup significantly decreased the tested parameters even at lower concentrations (≥ 5 μg/mL GLY-equivalent concentration). The adverse effects of GLY and Roundup were not detected in other studies on sperm DNA integrity (e.g., Nerozzi et al. 2020). Teratogenic effects of GBHs were observed in amphibians (Paganelli et al. 2010). In contrast, GLY was not lethal to embryos but did cause edemas at the highest concentration (50 mg/L) (Bonfanti et al. 2018). The possible teratogenic effect of GBHs was evident in primary embryonic stem cells of *D. melanogaster* (Argueta and Torres 2017). Preconceptional exposure to GLY (2 mg/kg) affected maternal ovarian function during mid- and post-gestation in female mice, while the reduced post-weaning number of ovarian secondary follicles and altered ovarian proteome were demonstrated (Novbatova et al. 2022), and it was assumed that GM crop-related exposure to GLY has been linked to significant adverse perinatal health effects (Reynier and Rubin 2025).

The endocrine-disrupting effects of GLY and GBHs were demonstrated by estrogen receptor and testosterone-disruptor effects, estrogenic effects, inhibitory effects on aromatase (a key enzyme in the biosynthesis of steroids), and inhibited biosynthesis of estradiol and testosterone (Romano et al. 2010; Thongprakaisang et al. 2013; Cassault-Meyer et al. 2014; Young et al. 2015; Mesnage et al. 2017; Muñoz et al. 2021). A nonmonotonic dual dose–response curve was evident in human thyroid cells exposed to Roundup Original DI from 6.5 to 6500 μg/L, with alternation between toxic and proliferative effects at different concentrations (Dal’Bó et al. 2022). The observed phenomenon is a well-known characteristic of endocrine-disrupting chemicals (Zoeller and Vandenberg 2015). In the JEG3 cell line, decreased aromatase activity by exposure to co-formulants (POEA and alkyl polyglucoside) and formulated GBHs including Roundup Classic, Roundup WeatherMax, Glyfos, was demonstrated at an 800-fold lower concentration than the case with GLY alone. GLY had effects only at 1/3rd of the agricultural dilution indicating the higher endocrine-disrupting effects of the formulations primarily caused by the presence of the co-formulants (Defarge et al. 2016).

GLY negatively impacts the female reproductive system by inducing oxidative stress, disrupting reproductive hormone regulation, causing histological alterations in ovarian and uterine tissues, and impairing ovarian function in both human cell lines and animal models (Stone et al. 2025). The hormonal effects of GBHs were evident in altered oxytocin secretion in bovine luteal cells (Wrobel 2018), as well as modified expression of estrogen-sensitive genes in exposed rats (Varayoud et al. 2017). GLY increased aromatase mRNA levels and decreased testosterone (Clair et al. 2012). Moreover, altered gonadotropin expression, hormone levels (estrogen, androgen, and progesterone), and follicle-stimulating and luteinizing hormone were observed (Romano et al. 2012; Perego et al. 2017; Wrobel 2018). Such effects indicate the need for refined risk assessment methods capable of detecting the consequences of disrupted endocrine functions and hormonal integrity (Vandenberg et al. 2012).

## Discussion

The occurrence of GBH residues in the environment, food, and food chains is now a global reality. The environmental fate of GLY and GBHs depends heavily on environmental factors, pH, temperature, microbial activity, and climatic conditions (Mamy et al. 2016; Grandcoin et al. 2017). The timing and frequency of GBH applications also drives environmental fate, exposures, and potential adverse impacts (Candela et al. 2010; Hébert et al. 2019). Considerable variability occurs in environmental concentrations of GLY. Analytical detection and quantification of GLY levels will continue to pose pragmatic challenges due to the poor solubility, chelating properties, and high polarity of GLY, in addition to the metabolism to AMPA (Gomes et al. 2017b). Consequently, developing analytical methods for the accurate and reliable detection of GLY and AMPA is crucial for conducting effective environmental and human health risk assessments (Valle et al. 2019; Singh et al. 2020).

Pesticide regulatory laws and polices are focused on minimizing general population risks arising from ingestion of pesticide residues in food and beverages. The collection of data needed to refine occupational and bystander risk assessments receives far less attention, as does evaluation of impacts on non-target organisms and the ecosystems supporting them (Brovini et al. 2021; Ahuja et al. 2024; Bartling et al. 2024; Evalen et al. 2024). Despite limited research on GBH co-formulants, their presence in products as applied can clearly alter GLY and GBH environmental fate and risk gradients (Mesnage et al. 2015; Li et al. 2016; Klátyik et al. 2017; Rodriguez-Gil et al. 2017). More effort is warranted in tracking and assessing co-formulant exposures and risk profiles across environmental matrices and over time.

A wide and dynamic range of biochemical/physiological modes of action can trigger adverse impacts, especially in the wake of heavy, frequent, and/or long-term applications of GBHs. The sensitivity of various microorganisms differs and depending on the mix of exposures to GLY, AMPA, and other pesticides (Mesnage and Antoniou 2020; Rainio et al. 2021). The intensive and long-term use of GLY results in the selection of certain bacterial and fungal strains, leading to alterations in the composition of microbial communities in soil, surface waters, plant surfaces, and animal intestinal tracts (Staub et al. 2012; van Bruggen et al. 2018). In addition, the endocrine-disrupting effects of GBHs on microorganisms in the gut can trigger adverse health effects in a myriad of ways (Swanson et al. 2014; Owagboriaye et al. 2021; Motta et al. 2022). Possible effects on human reproduction and increased risk of chronic disease remain worrisome and warrant the development of refined risk assessments methods (Rueda-Ruzafa et al. 2019; Hu et al. 2021; Puigbó et al. 2022).

In tandem with the calls for more holistic regulation, the “real-life risk concept” (RLRS) has gained momentum as a way to capture the combined hazards of concurrent exposures in realistic settings (Karzi et al. 2023; Dinca et al. 2023; Sevim et al. 2024). Traditional toxicological assessments typically evaluate a single active compound under controlled conditions; however, real-world exposures frequently involve multiple agents, whether additional pesticides, surfactants, or environmental pollutants (Karzi et al. 2022; Tsatsakis et al. 2019). This is the case for the new generation of GM food crops tolerant to multiple herbicides, such as GLY, dicamba and 2,4-dichlorophenoxyacetic acid (Cirstea et al. 2024; Docea et al. 2023; Nechalioti et al. 2023).

Studies have shown that even nominally “inert” additives in GBHs – such as POEA – can potentiate GLY’s penetration into cells, modify its biochemical targets, or produce independent toxicological effects. Moreover, exposure to co-occurring contaminants may exacerbate oxidative stress or disrupt microbiomes in ways that are not accounted for in single-chemical frameworks (Mesnage et al. 2021b). By highlighting these interactions, the RLRS underscores the need for composite evaluations that better reflect how populations, wildlife, and ecosystems encounter pesticide mixtures in everyday life (Tsatsakis et al. 2019).

## Conclusion

Based on published studies, common side effects following GBH applications include the disruption of the microbiome, neurotoxicity, cytotoxicity, reproductive, oncogenic, and teratogenic effects. New modes of action and impacts at several trophic levels continue to be discovered, many driven by the sheer intensity of GBH use. The potential health effects of the physiological processes affected by the newly identified specific inhibition or inactivation of αVβ3 integrin functions by GLY is an emerging challenge for those conducting GLY and GBH risk assessments. A larger share of the toxicological and ecosystem studies and assessments should focus on the environmental fate and toxicity of widely used formulated products, not GLY AI nor individual, stand-alone co-formulants.

## Data Availability

Not applicable in this specific case as this is a review article.
